# Due Diligence in International Alliances: Performance Outcomes and the Moderating Effect of Geographic Distance and Partner-Specific Experience

**DOI:** 10.1007/s11575-025-00604-5

**Published:** 2026-01-05

**Authors:** Catherine Elizabeth Georgiou, Nigel Driffield, Hossam Zeitoun

**Affiliations:** 1 School of Business and Management, Royal Holloway University of London, Egham, UK; 2 Warwick Business School, University of Warwick, Coventry, UK

**Keywords:** International alliance performance, Information asymmetry, Due diligence, Geographic distance, Partner-specific experience

## Abstract

There is a general assumption in the literature that due diligence is a vital ex ante step in alliance formation. However, the actual relationship between alliance performance and due diligence has not been empirically studied. Drawing on theoretical insights from information economics, we address this issue in international alliances, considering each party’s due diligence investigations. Demonstrating that due diligence is indeed beneficial for performance, we go a step further and show that this beneficial effect is negatively moderated where contextual factors affect the information environment. We find that both geographic distance which increases information asymmetries and partner-specific experience which decreases information asymmetries lessen the value generation potential of due diligence. This paper thus confirms the assumption in the literature and also introduces contextual limitations. This paper therefore helps address the cost–benefit scenario of due diligence.

## Introduction

Strategic alliances have attracted much attention in the management and international business fields, yet as Ryan-Charleton et al (2022) note, in the domain of alliance outcomes, there is still a lot to learn. Partner selection has been shown to play an important role in the value generation prospects of collaborative relationships (Mindruta et al, 2016; Robson et al., 2019). The literature has explored partner typologies in alliance portfolios for innovation outputs (Hagedoorn et al., 2018) and the capability to identify appropriate types of partners for alliance performance (Degener et al., 2018). Furthermore, in the international domain where country-specific factors and information asymmetries add layers of complexity to alliance relationships, extant scholarship (e.g. Chand & Katou, 2012; Dorobantu et al, 2020) confirms that the selection of partners is particularly important.

Information economics, which has been gaining increasing interest in the management field (McCann et al., 2016; Reuer & Sakhartov, 2021; Reuer et al., 2013; Xing et al., 2021), holds that exchange hazards in collaborative relationships can be addressed through the appropriate selection of exchange partners (Reuer & Devarakonda, 2017). This theoretical standpoint focuses on the role of information in the reduction of ex ante adverse selection concerns. A firm accesses information either passively, by receiving signals of quality sent by other firms for example via prominent affiliates (Reuer & Ragozzino, 2014), capability demonstration (Jolink & Niesten, 2021; Stern et al., 2014), and corporate social responsibility activities (Wang et al., 2023); or via active information search. There are two elements of active information search: intensive and extensive (Reuer, 2009). Intensive search involves searching for the range of partners within a decided criterion. The partner selection literature in general has considered the broader typologies or characteristics of partners firms seek, and furthermore, Reuer and Devarakonda (2017) have considered partner selection from an information economics perspective. Hence, extensive search has been explored in detail. However, intensive search, which refers to the efforts undertaken to evaluate specific partners, has received far less attention. Our main theoretical contribution thus lies in extending the application of information economics in the alliance field, by exploring intensive search.

Organizations perform intensive search via due diligence. While the alliance literature has been relatively silent on the role of due diligence for performance outcomes, due diligence is attracting increasing interest in the business and management (Guenther et al., 2022; Reuer & Sakhartov, 2021) and international business (IB) (Reuer & Sakhartov, 2021; Wilhelm, 2024) fields, where it has been identified as vital for overcoming the complexities of selecting specific partners for international alliances (Kano, 2018; Wang et al., 2023), though we are not aware of a study which tests this empirically. We address the aforementioned gap by investigating due diligence and performance outcomes in international alliances, which we define as cross-border contractual relationships among firms which are distinct legal entities.

Despite the fact that much of the literature discussed above highlights the importance conceptually of due diligence, and that its importance is widely mentioned in the practitioner literature (Boston Consulting Group, 2025; Engelbrecht et al., 2019), the importance of due diligence in explaining subsequent performance is largely missing from both the alliances and JV literature. Where it is mentioned, its importance is limited to the focal firm, but two issues are overlooked. The first is the extent to which due diligence extensiveness informs firm decisions and therefore subsequent performance, and the second is whether due diligence activities by each party are complements or substitutes. This is especially important given the finite resources of firms, and the high cost of due diligence, especially in the international domain. We address this gap by questioning the value of due diligence in the presence of contextual factors that alter the information environment. Specifically, we look at the potentially moderating role of geographic distance and partner-specific experience on focal firm due diligence. These factors affect the availability of information for the focal firm in different ways. Geographic distance affects the accuracy and availability of information that can be collected via due diligence; and partner-specific experience affects the information that the focal firm possesses prior to undertaking due diligence.

Due diligence is an activity associated with high levels of privacy and is therefore not detailed in publicly available databases. This potentially explains the lack of studies in this domain. We are able to offer novel insights into due diligence and performance because we have access to a unique database detailing the actual due diligence evaluations undertaken by each alliance partner, and the detail allowing us to directly measure the attainment of financial objectives. Our data derive directly from industry practitioners and have been collected over 15 years, from firms located in over 40 countries, thus also enabling us to extend the geographic scope of previous research. We find that due diligence is beneficial for alliance performance outcomes. However, this benefit is lessened as geographic distance increases, and in the presence of partner-specific experience.

This paper is structured as follows: first, we discuss our theoretical framework and develop our hypotheses. Then, we describe our data and method. Next, we present and discuss our results and offer concluding remarks.

## Theoretical Framework

Our explorations are based on theoretical arguments from information economics. We adopt this perspective because information asymmetries and subsequent adverse selection concerns are central to the formation and running of these alliances, giving rise to growing scholarly body of work exploring alliances from an information economic perspective (Jolink & Niesten, 2021; McCann et al., 2016; Reuer & Devarakonda, 2017; Wang et al., 2023). While this theoretical framework has been used to explore due diligence in the M&A literature (e.g., Reuer & Sakhartov, 2021), we specifically look at the alliance context because alliances present a different type of adverse selection problem. As noted by Reuer and Devarakonda (2017), the classic problem of Akerlof (1970) applies to alliances in the following manner; Party A does not possess all the relevant information on Party B, and Party B has motives for misrepresentation, which refers to the withholding, or even distorting information. As alliances are contractual collaborations rather than buyer–seller transactions; Party B also faces this problem.

Firms may engage in misrepresentation, for example by presenting resources or capabilities as higher quality than their true value for reasons including increased bargaining power in negotiations, or to encourage the other party to increase effort inputs. Information asymmetries between the partners also present challenges in assessments of partner fit – whether the partner is a good match on a range of relevant factors. It is therefore challenging to evaluate partner resources, aggravating adverse selection concerns (Reuer & Devarakonda, 2017). This is highlighted in the more resent work of Reuer (2024, p.268) where it is stated that “alliances are…shaped by information asymmetries and adverse selection risk.” This draws attention to the importance of intensive search. Due diligence reduces information asymmetries by collecting information which is neither publically available nor accessible. Given the cost of due diligence in both financial and effort inputs, this paper looks to demystify the circumstances in which due diligence is beneficial for performance outcomes, and the factors which affect the value generation prospects of due diligence. We consider the due diligence of each party (focal and partner firm) and explore the potential moderating effect of geographic distance and partner-specific experience.

## Hypotheses

### Due Diligence by the Focal Firm in Alliances

We begin with a somewhat intuitive argument about the role of due diligence for performance. In the assessment of partner viability, a firm decides on whether to undertake due diligence, and the extent of investigations. The extent of due diligence refers to the factors which a given firm investigates in the due diligence phase. For further discussion of this, see Brueller et al., 2018; Harvey & Lusch, 1995). Due diligence can be limited to the traditional (initial) stages of financial and legal factors, though extensive due diligence will gather additional data to reduce information asymmetries on a range of factors including softer elements such as cultural fit, and assessments of capabilities and resources (Gulati et al., 2012; Harding & Rouse, 2007; Jambulingam & Saxton, 2021; Russo & Cesarani, 2017). Macroinvestigations may also be included, as these offer a more complete picture of the potential transaction (Harvey & Lusch, 1995; Lahiri et al., 2021; Lawrence, 2024).

Effective screening requires a spectrum of factors (Lawrence, 2024), to identify misrepresentation and partner fit. Partner misrepresentations of resource quality and capabilities can have a detrimental effect on the focal firm’s ability to capture value from the alliance. Additionally, it is important to look for partner fit, to ensure the alliance is apt to maximize value generation potential. Partner fit has been identified as an important factor in maximizing alliance benefits (Lahiri, 2021), as this promotes alliances with partners that are more appropriate, or at least those perceived as so at the outset of the relationship. According to Wong et al (2005), even the perception of partner appropriateness is sufficient to have a positive effect on performance in international collaboration. The reduction of adverse selection concerns will increase effort and other investments into the relationship. We thus posit that as due diligence extent increases and information is collected on the breadth of factors required for a holistic reduction of information asymmetries and adverse selection concerns, focal firm performance will likely improve.

#### Hypothesis 1 (H1)

More extensive due diligence by the focal firm will improve focal firm performance.

### Due Diligence by the Partner Firm in Alliances

A key difference between the buyer–seller type transactions of acquisitions, and the contractual alliance relationship, is that in acquisitions, it is only the buyer who undertakes due diligence to reduce adverse selection concerns. As there is more work on due diligence in this field, the one-sided perspective of due diligence is taken as the status quo (see for example Reuer & Sakhartov, 2021; Wang et al., 2020; Wangerin, 2019). However, owing to the contractual nature of alliances, it is possible that either party can asses partner fit and misrepresentation. The key difference between these is that assessments of misrepresentation only benefit the firms undertaking the due diligence, whereas partner fit can be assessed by the partner firm, and benefit the focal firm also. The focus of intensive search is to identify whether the goals and key attributes of the partners align. One can comfortably assume that where the goals and attributes of Firm A align with those of Firm B, the reverse will also apply—the goals and attributes of Firm B will align with those of Firm A.

We posit that in alliances, issues of partner fit dominate adverse selection concerns, and therefore assert that regardless of which partner undertakes due diligence, if due diligence is extensive to cover the breadth of relevant factors, both partners will benefit. Partner fit promotes better alliance outcomes (Ryan-Charleton et al., 2022) as partners benefit one another in pursuit of their own goals. As Wong et al., (2017, p.821) state, partners “with cooperative goals not only promote their own goals…they also contribute to their teammates success.” Hence, where the partner undertakes extensive due diligence, not only do they alleviate their *own* concerns about goodness of fit, they simultaneously alleviate the concerns of the focal firm, suggesting that the performance outcomes of the focal firm will benefit from due diligence despite not having invested in due diligence themselves, providing the partner has done so.

#### Hypothesis 2 (H2)

More extensive due diligence by the partner firm will improve focal firm performance.

### The Moderating Effect of Geographic Distance

This paper has thus far argued for the beneficial effect of alliance due diligence, which is inline with the current academic and practitioner literature. However, owing to the financial and effort investment costs inherent to due diligence, we now extend the conversation to consider the circumstances in which the value maximization potential of due diligence may lessen. We begin with a consideration of geographic distance between the partners.

Technological advancements in the modern era facilitate interactions across vast geographic distances (Troise et al., 2023). However, geographic distance continues to pose challenges to interfirm activity (Kok et al., 2020; Tower et al., 2021). As such distance heightens information asymmetries (Chen et al., 2022), one can reasonably assume that greater distance between alliance partners will require more extensive due diligence. However, a key premise of information economic theory is efficiency. The value of due diligence is therefore dependent upon whether the firm can generate a solid understanding of the relevant attributes of the transaction partner. Reuer’s (2009) work on information economics argues that the ability of firms to understand the relevant attribute of transactions shapes the geographic distribution of economic activity. Hence, while it becomes more challenging to gather information, the need for this information simultaneously amplifies, aggravating adverse selection concerns (Reuer & Lahiri, 2014). The question is therefore, whether the value generated by due diligence holds, as physical distance between alliance partners increases. Or, do the increased complexities of geographic distance reduce the value of information gathered from due diligence, thus decreasing its value for performance outcomes?

According to Reuer (2009), hard information can be evaluated effectively even in the context of large geographic distances. This suggests that the traditional stages or less extensive due diligence will be only minimally impacted by distance. However, it is well recognized that the softer factors of more extensive due diligence are incredibly important for value creation potential in alliances as it is these factors which determine whether organizations will mesh (Harding & Rouse, 2007; Liu et al., 2021). These soft factors tend to be “geographically localized” (Reuer, 2009, p.248; Zhao, 2021) owing to their tacit nature (Atakhan-Kenneweg et al., 2021), it is thus more challenging to obtain accurate information on geographically distant alliance partners (van Kranenburg et al., 2014). In simple terms, the quality of the information that is gleaned from extensive due diligence diminishes with distance. This is consistent with the large literature on international knowledge flows, and indeed with the wider literature on the theory of the multinational. It is also consistent with the wider literature on alliances that employs the frameworks from information economics. For example Reuer and Lahiri (2014) note that geographic distance can hamper alliance formation as large distances make the evaluation of partner resources and prospects more problematic. We therefore posit that the beneficial effect of extensive due diligence on alliance performance will lessen as geographic distance increases.

#### Hypothesis 3 (H3)

The beneficial effect of extensive focal firm due diligence will lessen as geographic distance between the partners increases.

### The Moderating Effect of Partner-Specific Experience

We continue the conversation about the factors influencing the value of due diligence by considering the role of partner-specific experience. We define this as “experience accumulated from repeated allying with the same partner” (Holloway & Parmigiani, 2016; Wang et al., 2022, p.413). According to Wang et al. (2022) and Gulati et al. (2009), explorations into these repeated partnerships will enrich our understanding of alliance outcomes. In doing so, we draw on the concept of redundant information, which has roots in economic sociology (Burt, 2002; Uzzi, 1996). While at least to our knowledge, this is yet to be applied to the information economics field, the concept of redundant information has offered fruitful insights in the literature on alliance portfolios and networking (Arora et al., 2021; Bi et al., 2022). We follow the seminal work of Uzzi (1996) and the more recent work of Bi et al. (2022), to define redundant information as information accessed which is the same or so similar that it does not offer any significant novelty or additional benefits to the firm. This allows us to question whether intensive search meets the efficiency criterion of information economic theory.

The practical application of information efficiency is clarified in the seminal work of March and Simon (1958), where it is highlighted that information search and gathering are undertaken only to the extent that the costs incurred are outweighed by the benefits. With partner-specific experience, information is gradually accumulated from interactions. If some of the information collected by due diligence is already possessed this information will be redundant, as information asymmetries will have been reduced prior to undertaking due diligence. It is noteworthy that as firms respond to ever-changing situations in their internal and internal environment, base-level due diligence such as financial and legal factors may change in the time between interactions, and therefore require investigating. However, soft factors such as whether the other party’s organizational culture is compatible with their own, are suggested to be more stable or gradually evolving (Sull et al., 2022), suggesting that information gathered from the extensive levels of due diligence may be redundant.

The idea of partner-specific experience resulting in the accumulation of knowledge about soft factors is evident in extant alliance scholarship. Lioukas and Reuer’s (2015) find that experience with a specific alliance partner leads to familiarity with behavioral patterns, facilitating the calculation of when self-interested activity may occur, and how to best navigate interactions. Additionally, Li et al. (2008) noted that even minimal prior ties with the alliance partner are sufficient to understand the routines, processes, and organizational culture. We therefore posit that that the beneficial effect of extensive due diligence on alliance performance will lessen as partner-specific experience increases.

#### Hypothesis 4 (H4)

The beneficial effect of extensive focal firm due diligence will lessen in the presence of partner-specific experience.

**Fig. 1:**
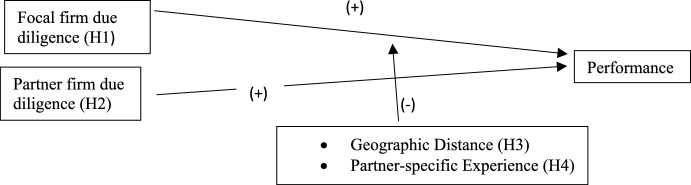
Due diligence and alliance performance

Figure 1 summarizes our arguments by depicting the proposed relationship between focal and partner firm due diligence and alliance performance. We suggest that both focal firm due diligence (Hypothesis 1) and partner due diligence (Hypothesis 2), will have a positive effect on focal firm performance, and that the beneficial effect of focal firm due diligence will be negatively moderated by geographic distance (Hypothesis 3) and partner-specific experience (Hypothesis 4).

## Method

### Sample

We required detailed information on due diligence and performance outcomes. Owing to the high levels of secrecy associated with due diligence, information about this activity is not held in publically available databases. The performance-related information we required is also not available in such databases. Ryan-Charleton et al (2022) noted that scholarship on alliance performance relies on either proxies (e.g., managerial satisfaction or partner commitment), or on financial archival databases (e.g., stock market reactions or accounting data), though this presents difficulties “with disentangling alliance-related contributions to non-alliance-related contributions” (p.734). Hence, the point of Oxley (2009), that firm-level measures of performance do not readily apply to alliances, holds relevance even today. It is thus suggested that alliance research utilize *non-traditional data* held by the firms (Choi & Contractor, 2016; He et al., 2020; Ryan-Charleton et al., 2022). This is inline with Rugman’s advocacy of firm-level data deriving directly from practicing managers (Casson, 2016; Narula & Verbeke, 2015).

We use a unique dataset of international alliances, compiled by the alliance consultancy firm Alliance Best Practice (ABP) Ltd, as part of a longitudinal study to best advise their clients on maximizing alliance outcomes. ABP have given the authors exclusive access to these data which span 40 countries and were collected over a 15-year period (2000–2016). Hence, we have far richer data than we could have feasibly collected via our own survey (the only alternative to address our aims).

Prior to devising the data collection instrument, ABP undertook a focus group of 80 alliance executives, the aim of which was to understand the most important factors for alliance success. The focus group provided qualitative input based on the following question: “what makes an alliance successful?” The information was coded (thematically) by ABP by one individual to ensure consistency, and the most commonly re-occurring themes were identified. The aim of the data collection instrument was to gather information on these themes. Before the data collection, instrument was officially used to generate the database which the authors utilized in this study, a pilot study was undertaken by ABP, for validation purposes. The pilot study was performed on 340 members of the Association of Strategic Alliance professionals. Members were from various countries, ensuring the suitability of constructs for an international sample. This practice is recommended in Hult et al (2008) as one of the most reliable methods for establishing data equivalence for cross-border scholarship.

The data collection instrument captured quantitative data about specific international alliance relationships. The data were collected by ABP utilizing their international network of alliance professionals. The data were collected face-to-face, over the phone, and via email, all by the same individual. The respondents are key persons involved in the alliance. Considering the wide geographic dispersion of the sample and the busy schedules of alliance professionals, this was the most feasible option. As the respondents are experts in the field, its is reasonable to assume that terms are understood, though explanations were provided.

We, the authors, undertook further verification steps.^1^ We checked the due diligence categories, and the measure of financial outcomes were reflected in the literature. For due diligence, as we found no extant scholarship detailing extent we drew on the closely related M&A literature, which is suited because “alliances are external corporate development activities, or means of inorganic growth of restructuring that can be compared with acquisitions (Reuer, 2024, p.268).” Although this literature did not empirically test due diligence, we confirmed the order of extent categories (e.g. Ahlstrom et al., 2014; Harding & Rouse, 2007; Harvey & Lusch, 1995;). For alliance performance, we confirmed our measure of revenue-based performance drawing on Christoffersen et al (2014). To verify there is no systematic bias in our data we compared the breakdown of sectors across those of the most-known commercially available alliance data, the Thomson Financial Securities Data Corporation (SDC)/Refinitive Platinum database, and found no significant differences in the distribution of observations across sectors. We also examined the temporal patterns of alliance formation and did not detect differences.

The dataset contains information on 52 relevant factors of alliance relationships. We utilized a subset which collected information on due diligence and performance (see Appendix). Furthermore, as our research aims required details on the due diligence activities of both partners in a dyad, we limited entries to those where both parties of the same alliance had responded. We have a final sample of 470 observations. As ABP utilized their client network to collect the data, they have detail on the alliances of many of the largest multinational organizations worldwide (a full list of companies is available in Nevin, 2014). It is noteworthy that the data goes beyond 2014, as ABP continued to collect data after the book was published, though the post-2014 entries are additional alliances, not additional firms, hence the list remains accurate.

### Dependent Variable

In line with a common suggestion in the literature (e.g., Charleton et al., 2022; Oliveira & Lumineau, 2019; Salvato et al., 2017), that scholars clarify outcome type, we focus on the attainment of revenue-based objectives. According to Christoffersen et al (2014), where financial performance is part of the goals or objectives of a firm, this measure reflects a combination financial and operational performance. This variable is measured on a five-point rank-ordered Likert scale ranging from *nowhere near* to *target exceeded* (see Appendix for more detail).

### Independent Variables

We begin with an explanation of our measures of due diligence extensiveness. As there is a lack of due diligence focused papers in the alliance literature, we draw on the few papers in the M&A field which detail this construct. Empirical or economic-modeling based studies have relied on proxies such as advisory fees (Reuer & Sakhartov, 2021) and number of days (Wangerin, 2019), because of the lack of available data on actual due diligence investigations. However, advisory fees can be inaccurate because fees can vary widely depending on the consultants selected, for example often firms select smaller or boutique advisors which cost up to three times less than their larger counterparts (Song et al., 2013). Additionally, the advisory fee proxy will be less reliable in the international domain because different national markets can be expected to have different pricing. Furthermore, Reuer and Sakhartov (2021) quote Axial’s Chief Executive Officer who states that “a lot of due diligence can be done yourself these days” (p.4) suggesting that firms try to keep costs down where feasible. Following a similar line of thinking, number of days can also be inaccurate because firms can be generally assumed to aim for efficiency by getting as much done in as little time as possible. Moreover, advisory fees and number of days cannot capture the specific factors investigated in due diligence.

Extant work that has discussed how due diligence is undertaken, highlights that the factors investigated are not in a haphazard order, but sequential (Ahlstrom et al., 2014; Brueller et al., 2018; Harding & Rouse, 2007; Harvey & Lusch, 1995; Marks & Mirvis, 2015). Harvey and Lusch (1995) clearly outline that due diligence begins with what they call traditional, or hard factors that focus on measurable financial performance, and legal issues that may be subject to contractual arrangements. Harvey and Lusch (1995) noted that due diligence can the be extended to encompass soft factures such as organizational culture and can extend further to considerations to macrorelevant factors. This perspective is supported in the more recent literature (e.g., Ahlstrom et al., 2014; Brueller et al., 2018; Harding & Rouse, 2007; Lahiri et al., 2021; Lawrence, 2024; Marks & Mirvis, 2015) and forms the basis of our measurement of due diligence extensiveness*,* which begins with hard internal factors and progresses to soft external and macro relevant factors (see Appendix for more detail).

Our variables *focal firm due diligence extensiveness*, and *partner firm due diligence,* both derive from the same question in our data collection instrument (see the second question in the Appendix), and are thus measured in the same way. *Focal firm due diligence extensiveness* is the response of the firm upon which we focus our analysis; *partner firm due diligence extensiveness* is the partner of the focal firm in the given alliance. We are able to do this because in our data we have, and can identify, the responses of both firms in a dyad. This approach has been used by Li and Reuer (2022) in their examinations of focal and partner firm characteristics in international alliances.

Our due diligence extensiveness variables are measured on a five-point rank-ordered Likert scale ranging from *no due diligence* to *full and complete due diligence* (see Appendix for more detail). Additionally, respondents had the option to point out if their due diligence practice did not fit the given ordering, although this was not the case for any of our respondents.

The variables we test for moderation effect are geographic distance, and partner-specific experience. *Geographic distance* is calculated using the CEPII GeoDist database (Mayer & Zignago, 2011). Bilateral distance between home and partner nations is calculated in kilometers between the largest city (weighted by population of inhabitants as a proportion of the national population) in each country. This reduces concerns of calculated distance between particular cities/points which can be distorted when considering larger economies (Castellani et al., 2013). As is suggested in Wang et al (2022) and Gulati et al (2009), we measure *Partner-specific experience* by the amount of previously formed alliances with the same partner. We use a 5-point Likert scale ranging from *zero* to *many* (see Appendix for more detail).

### Control Variables

*Alliance experience* measures the extent to which the focal firm engages in alliance relationships in general; product/service *complementarity* measures complementarity versus substitutability, a key driver in the selection of partners (Mindruta et al., 2016). *Strategic importance of the relationship* and *relevance of partner market position* are from the focal firm perspective to measure strategic relevance. These alliance level controls are measured on a 5-point ordered Likert scale, with higher scores denoting a greater the level of a given variable (see Appendix).

*National cultural distance* is measured using Ghemawat’s (2007) language, diaspora, and religion matches, building on Tung and Verbeke (2010) who point out that factors such as religion and diaspora could affect the actions of people in any nation. We do not use Hofstede’s (1980) dimensions because according to Kostova et al (2020), these portray informal institutional distance. *Law and order distance* is relevant because of the contractual basis of alliances, and because Roy and Oliver (2009) noted that the legal environment is a telling variable in international collaboration. We measure this from the ICRG^2^ database, which uses political, financial, and economic data. Countries are given scores based on expert opinions. Dummy variables control for year effects, industry, firm type (MNE, MNE subsidiary, and standalone business), and home country.

### Statistical Model

Our data were collected over a long time-scale (15 years), though this is not panel data. As such, we treat our data as cross-sectional. We use an ordered probit regression as used by Reuer and Ragozzino (2012) for variables of incrementally increasing values. The suitability of this type of regression over the more commonly used linear regression is explained in Basile et al (2003), where it is noted that in a linear regression a variable with a value of 2 would be assumed to have twice that of a variable with a value of 1; however, an ordered probit model has no such presumption of cardinality; hence, the value of 2 is simply more than that of 1. This logic applies to our variables; hence, we rely on an ordered probit regression. We explain our basic model below.

The basic model is:

Performance = f (Due Diligence Partner, Due diligence, Due Diligence*Alliance_Experience, Due Diligence*Geographic Distance, controls_i)))_ + ${\epsilon }_{i}$).

## Results

Tables 1 and 2 report the descriptive statistics and correlation coefficients. The most common industries are pharmaceuticals (47.41%) and information technology/computing (42.22%). To verify there is no systematic bias in our data, we compared the breakdown of sectors across those of the most-known commercially available alliance data—Thomson Financial Securities Data Corporation (SDC)/Refinitive Platinum database. There are no significant differences in the distribution of observations across sectors in our data compared with these. We also examined the temporal patterns of alliance formation and did not detect differences across the two data sources.

**Table 1:** Descriptive statistics

Variable	Obs	Mean	Median	SD	Min	Max
Performance	470	2.929	4	1.342	0	4
Due diligence	470	2.631	3	1.479	0	4
Partner due diligence	470	2.604	3	1.534	0	4
Law and order (focal)	470	5.157	5	0.502	2.5	6
Law and order (partner)	470	5.143	5	0.493	2.5	6
National culture	470	2.717	3	0.592	0	3
Geographic distance	470	2077	0	3736	0	14,418.6
Relevance market position (focal)	470	1.866	2	1.629	0	4
Relevance market position (partner)	470	1.614	2	1.480	0	4
Complementarity (focal)	470	2.491	3	1.462	0	4
Complementarity (partner)	470	2.103	3	1.753	0	4
Strategic importance (focal)	470	1.642	2	1.484	0	4
Strategic importance (partner)	470	1.614	2	1.440	0	4
Alliance experience (focal)	470	2.008	2	1.324	0	4
Alliance experience (focal)	470	1.992	2	1.332	0	4
Partner-specific experience	470	1.233	1	1.400	0	4

**Table 2:** Correlation coefficients

Variable	1	2	3	4	5	6	7	8	9	10	11	12	13	14	15
1 Performance	1														
2 Due diligence	0.50	1													
3 Partner due diligence	0.43	0.58	1												
4 Law and order distance	− 0.02	− 0.01	− 0.02	1											
5 National culture	0.18	0.29	0.29	0.11	1										
6 Geographic distance	− 0.10	− 0.24	− 0.21	− 0.00	0.76	1									
7 Relevance of market position (focal)	− 0.16	− 0.20	− 0.28	0.00	0.00	− 0.01	1								
8 Relevance of market position (partner)	− 0.18	− 0.29	0.20	− 0.01	− 0.01	− 0.01	− 0.27	1							
9 Complementarity (focal)	− 0.13	− 0.15	− 0.19	− 0.21	− 0.02	− 0.07	0.03	0.20	1						
10 Complementarity (partner)	− 0.14	− 0.18	− 0.15	− 0.07	− 0.07	0.03	0.11	0.21	0.10	1					
11 Strategic importance (focal)	− 0.17	− 0.26	− 0.19	− 0.09	− 0.06	0.05	− 0.04	0.27	0.14	0.28	1				
12 Strategic importance (partner)	− 0.15	− 0.22	− 0.25	0.07	− 0.08	0.06	0.15	0.26	0.01	0.27	0.15	1			
13 Alliance experience (focal)	− 0.05	− 0.11	− 0.04	− 0.03	0.06	− 0.04	0.25	0.09	0.16	0.02	0.24	0.09	1		
14 Alliance experience (partner)	− 0.05	− 0.03	− 0.11	0.01	− 0.01	− 0.03	0.09	0.26	0.00	0.19	0.10	0.25	0.15	1	
15 Partner-specific exp	− 0.14	− 0.24	− 0.15	0.02	− 0.05	0.02	0.20	0.09	0.17	0.01	0.33	0.13	0.19	0.2	1

The data show that for performance in terms of revenue, the mean is 2.646 and the median is 3, the second highest category. This shows that a majority focal firms in the sample achieve their revenue aims. The mean for focal and partner firm due diligence is 2.631 and 2.604, respectively, the median for both is 3. Most correlation coefficients among independent/control variables are well below 0.4, so multicollinearity is an unlikely concern. However, infrequent exceptions among control variables prompted the testing of variance inflation factors. Explanatory variables are well below 10 hence multicollinearity is not an issue.

The ordered probit regression results (Table 3) demonstrate that undertaking a greater extent of focal firm due diligence has a positive effect on performance outcomes (*p* < 0.001) supporting Hypothesis 1. The results also show that more extensive partner firm due diligence does benefit focal firm, though this falls below significant thresholds. Hence, this paper does not find statistical support for Hypothesis 2.

**Table 3:** Ordered probit regression analysis

Variables	Performance (objectives)	*p value*
Due diligence	0.461	0.000***
	(0.086)	
Partner due diligence	0.066	0.189
	(0.051)	
Due diligence*geographic distance	− 0.000	0.001***
	(0.000)	
Due diligence*partner-specific experience	− 0.153	0.000***
	0.033	
Complementarity (focal)	− 0.042 (0.136)	0.758
Complementarity (partner)	− 0.183 (0.136)	0.178
Relevance of partner market position (focal)	0.245 (0.156)	0.116
Relevance of market position (partner)	− 0.011 (0.154)	0.945
Law and order distance	0.007 (0.172)	0.965
National Culture	0.394 (0.189)	0.038
Geographic distance	0.000 (0.000)	0.058*
Strategic importance (focal)	− 0.006 (0.186)	0.963
Strategic importance (partner)	− 0.148 (0.189)	0.800
Alliance experience (focal)	− 0.191 (0.194)	0.324
Alliance experience (partner)	0.023 (0.193)	0.232
Partner-specific experience	− 0.194 (0.093)	0.315
Observations	470	
Pseudo R2	0.272	

All estimations include year dummies, industry dummies, country dummies, and firm type dummies

Standard errors are in parentheses, *p < 0.10, **p < 0.05, ***p < 0.01

We ran interaction terms to test whether the relationship between focal firm due diligence and performance is moderated by geographic distance and partner-specific experience. We find that greater geographic distance lessens the value of focal firm due diligence for performance (*p* < 0.001). Partner-specific experience also lessens the value of focal firm due diligence (*p* < 0.001). Both geographic distance and partner-specific experience thus negatively moderate the beneficial effect of extensive due diligence; Hypotheses 3 and 4 are therefore supported.

We now turn to the marginal effects by category of alliance performance for our significant results (Table 4). A one-unit increase in focal firm due diligence extensiveness increases the probability of being in the highest performance category by 12 percentage points (*p* < 0.05); the second highest is insignificant, reduces the chance of being in the middle category by 1 percentage point (*p* < 0.05); in the second lowest category by 4 percentage points (*p* < 0.001); and in the lowest category by 75 percentage points (*p* < 0.05).

**Table 4:** Marginal effects for alliance performance

Variables	Category	Model 1	*p*
Due diligence	Category 1	− 0.754	0.009
	Category 2	− 0.036	0.000
	Category 3	− 0.013	0.003
	Category 4	0.003	0.162
	Category 5	0.121	0.000
Due diligence *Geographic distance	Category 1	5.38e-06	0.001
	Category 2	2.56e-06	0.002
	Category 3	9.60-e07	0.020
	Category 4	− 2.8e-07	0.192
	Category 5	− 8.67e-06	0.001
Due diligence*Partner-specific experience	Category 1	0.0206	0.000
	Category 2	0.0098	0.002
	Category 3	0.0037	0.006
	Category 4	− 0.009	0.188
	Category 5	− 0.0331	0.000

We have also tested the marginal effects for our moderator variables. Interacting focal firm due diligence with geographic distance showed very small changes to the marginal effects, and these changes were significant for each category besides the second highest.

For focal firm due diligence interacted with partner-specific experience, a one-unit increase in due diligence extensiveness reduces the probability of being in the highest performance category by 3 percentage points (*p* < 0.001); the second highest is insignificant; increases the likelihood of being in the middle category by 4 percentage points (*p* < 0.05); in the second lowest category by 10 percentage points (*p* < 0.05); and in the lowest category by 2 percentage points (*p* < 0.001).

To confirm our results are robust, we begin with the test for endogeneity with probit models of Naghi et al (2022), an update of the more familiar tests (e.g., Heritier & Ronchetti, 1994, Karaca-Mandic & Train, Karaca-Mandic & Train, 2003). The null of exogeneity is not rejected. While the endogeneity tests do not suggest a problem, endogeneity tests within maximum likelihood regressions have famously low power (Karaca-Mandic and Train, Karaca-Mandic & Train, 2003), so we also adopt an instrumental variable approach. The selection of instruments is crucial here, and no variable individually passed the test for instrument validity (Kitagawa, 2015). In order therefore to generate a valid instrument for both focal and partner due diligence, we regressed focal and partner firm due diligence separately as dependent variables against a vector of firm and dyad specific variables.^3^ We then obtain the fitted value from this regression and employ this value as the instrument for focal and partner firm due diligence. The instrumental variables (IV) estimation yielded very similar results, suggesting endogeneity is not a concern.

## Discussion

The academic (Kano, 2018; Wang 2023) and practitioner (Boston Consulting Group, 2025; Engelbrecht et al., 2019) literatures have focused on the benefits of due diligence. However, the value of alliance due diligence had not been empirically examined. Our paper has drawn on a unique dataset allowing us to address this gap by testing the relationship between focal and partner firm due diligence extent and alliance performance. We have done so by taking an information economics approach, which considers the reduction of information asymmetries between the partners in intensive search. Our results show that as the academic and practitioner literature suggest, due diligence is indeed beneficial for alliance performance, as we find that more extensive focal firm due diligence improves financial outcomes. However, considering the efficiency criterion of information economics, we delved deeper into the context of due diligence by investigating the factors which could lessen the beneficial effect of focal firm due diligence on performance. This fine-grained analysis showed that the beneficial effect of due diligence is negatively moderated by geographic distance between the partners and experienced with the specific alliance partner. While both of these factors impact the information environment, they do so in different ways. Geographic distance increases information asymmetries while causing challenges in obtaining accurate information; and partner-specific experience reduces information asymmetries via previous interactions.

We find that due diligence (undertaken by the focal firm) is less apt to address the need for information, as geographic distance increases. This adds to the literature suggesting that despite technological advances, geographic distance continues to influence collaborative transactions (Kok et al., 2020; Tower et al., 2021). We go a step beyond the consideration of the increased costs associated with distance, and demonstrate that the efficacy of focal firm due diligence diminishes as distance increases. Furthermore, as we capture the different types of information collected at different levels of extent, we are able to show that the sensitivity of this information to physical distance, depends on the type of information collected; for example, the financial and legal information collected at the less extensive levels is less affected by distance than the soft information collected in more extensive due diligence. It would be valuable for future scholarship to explore the ways in which firms address issues of geographic distance. One option might be to rely on information from the firm’s network. As Aggarwal (2020) notes, partner and partners-of-partners can be a way to access vital information. Signaling theory (Spence, Spence, 1974) might also offer some interesting insights, as signaling offers an alternative to intensive search. Here, the burden is on each partner to send costly signals to demonstrate unobservable attributes, with can be understood by the other firm. Interestingly, recent work has demonstrated that signaling is costlier than previously thought (DesJardine, 2021) and each firm would require a cost–benefit analysis of whether it is efficient to signal.

Our paper also adds to the growing literature which considers the role of partner-specific experience on alliance outcomes (Gulati et al., 2009; Wang et al., 2022). We find partner-specific experience, reduces the beneficial impact of focal firm due diligence because a significant proportion of information gathered from previous alliances with the partner, will overlap with information gathered via due diligence, and will thus be redundant. Redundant information will mostly be relevant to the more extensive levels of due diligence. This suggests that bearing the additional costs of extensive due diligence will be inefficient. This is to our knowledge, first attempt to consider redundant information from an information economics perspective, and has therefore made inroads into considerations of efficiency in information gathering and raises new questions. For example, does experience with a specific alliance partner render other types of information redundant? Does being in a central position in a network of relationships generate information which overlaps with that collected in due diligence? While these questions were beyond the scope of our paper, they will no doubt generate fruitful insights should they be explored in future scholarship.

Our analysis also emphasizes the importance of taking a nuanced view of due diligence. Our measure for due diligence goes beyond a simple dummy variable indicating the presence or absence of this activity; and also goes beyond the approach of taking proxies (see Reuer & Sakhartov, 2021). Instead, we apply the breadth of investigations, i.e., whether firms go beyond the financial/legal factors to further include softer elements and macro factors. We show that exercising focal firm due diligence in a wider range of facets is relevant for firm performance.

Our study is not without limitations. Firstly, we are looking only at financial objectives, though firms may have non-financially motivated reasons for forming an alliance with a specific partner. While this was out of the scope of our study we encourage future research to investigate the relationship between due diligence and non-financial objectives. Secondly, while the unique dataset enabled us to provide novel insights, the dataset was not initially collected for academic purposes but for the practitioner literature (see Nevin, 2014). The information contained in this dataset is what allowed us to access information on due diligence which is difficult to gather owing to the confidential nature. Furthermore, the data is comprehensive and contains many vital nuances, as well as being collected over a particularly long period (15 years). We recommend that future research complements our findings by undertaking primary data collection, for example, through survey type studies and qualitative interviews. Our data goes up to the year 2016, while we do not anticipate any large differences in more recent alliances, it would be interesting for survey data to look at more recent alliances. Another limitation is that we do not have access to details on the actual cities in which the firms in our database out located, we therefore rely on geographic distances in accordance with the bilateral distances between the largest cities between two countries in the Mayer and Zigano CEPII database (2011). While this approach is common (e.g., Arikan & Shenkar, 2013; Tan et al., 2019), future research could complement our findings via surveys identifying the city in which each firm is located. Finally, we focused on the value generation of due diligence for the focal firm; thus, the contextual moderatiors of geographic distance and partner-specific experience on the role of partner due diligence were out of the scope of our study. However, it would be interesting for future scholarship to undertake such explorations.

## Conclusion

This paper has offered insights into the role of due diligence for alliance performance, via an information economics lens. Our findings have important implications for practice, as we directly test the role of due diligence for financial performance. We demonstrate that focal firm due diligence should be extensive to maximize the value creation potential, by reducing information asymmetries on the range of relevant factors. Furthermore, as due diligence is inherently costly, it is also important that managers are aware the circumstances in which this activity may be less effective. We believe that this paper will encourage managers to undertake a cost benefit analysis of whether to undertake due diligence and the extent of investigations. In addition, they should consider both hard and soft information when planning for the due diligence process. We hope work helps bring out greater nuance that can guide alliance practitioners in managing the alliance formation process.

The data were supplied by Alliance Best Practice under a confidentiality agreement.

## Appendix

### Data Collection

Key details: firm name, partner name, location (country), partner location (country), size (number of employees), and alliance start date.

How close are you to obtaining your revenue-based objectives from the alliance?*

- Nowhere near
- Growth stage may not be achieved
- Stability achieved
- Stability achieved over time
- Target exceeded

Have you conducted due diligence for suitable alliance partners and if so what criteria were used?*

- No due diligence
- Hard aspects internal (examples include cash, technology, inventory, patents/trademarks)
- Hard aspects internal and external (in addition to the above, examples include market share and distributor/supplier contracts)
- Hard and soft elements limited focus: (in addition to the above, example include quality of management/human resources, customer loyalty, corporate culture)
- Full and complete due diligence: (in addition to the above, there is an inclusion of partner responses to macrolevel changes.

How many previous alliances do you have with the partner (include ongoing relationships)?*

- None
- A few
- Some
- Quite a lot
- A lot

What is your firm’s experience with alliances?*

- No experience
- Very little experience
- Some experience
- Experienced
- Very experienced

What is the strategic importance of this relationship?*

- None
- Very little
- Some
- Important
- Very important

What is the relevance of the partner market position?*

- None
- Very little
- Some
- Relevant
- Highly relevant

To what extent do your products/services/resources overlap with those of the partner?*

- A lot of overlap
- Some overlap
- A little overlap
- Very little overlap
- No overlap

*Questions have a space for other comments where respondents can make a different responses if for example neither of the categories apply.

## Footnotes

1 As we, the authors, were not involved in the data collection process, we treat the database as secondary data and carried out checks for representativeness of the sample, and checked for any obvious errors.

2 While there are numerous datasets that capture different aspects of institutional quality, our preference is the ICRG (see Howell 2013). This seeks to develop a data set that is targeted at informing firms’ internationalization decisions, rather than reflecting different countries foreign policy stances, or perspectives developed from particular ideologies. This has been previously used in multiple papers in the business and economic fields (see Driffield et al., 2016; Duncan, 2014).

3 These variables include: age, size, firm type and sector, and dyad level variables including measures of product complementarity, respective market position, and the strategic importance of the relationship. In addition, we include measures of both institutional and geographic distance.
